# Regulation of Energy Metabolism and Lipid Metabolites by IMP3 in Cervical Cancer

**DOI:** 10.3390/cimb47121014

**Published:** 2025-12-04

**Authors:** Xiaojun Lei, Wenyuan Yang, Sen Xu, Haigang Wu

**Affiliations:** 1 Xinyang Vocational and Technical College, Xinyang 464000, China; lxj_120@126.com (X.L.); wy.yang@henu.edu.cn (W.Y.); senxu@henu.edu.cn (S.X.); 2 School of Life Sciences, Henan University, Kaifeng 475004, China

**Keywords:** IMP3, oxygen consumption rate, energy metabolism, lipid metabolism

## Abstract

Cervical cancer outcomes remain suboptimal, underscoring the need to define molecular drivers that can be therapeutically exploited. Insulin-like growth factor 2 mRNA-binding protein 3 (IMP3) has been implicated in tumor biology, but its role in cellular metabolism is not well characterized. Here, we investigate the metabolic consequences of IMP3 suppression in HeLa cells and integrate these findings with transcriptomic analyses of clinical datasets. siRNA-mediated knockdown of IMP3 reduced mitochondrial oxygen consumption rate and altered cellular energy status, as evidenced by changes in ATP/ADP and NADP+/NADPH ratios, alongside shifts in key intermediary metabolites. Complementary bioinformatic analyses of Gene Expression Omnibus datasets revealed that cervical cancers with high IMP3 expression exhibit coordinated deregulation of metabolic, cell-cycle, and inflammatory response pathways relative to normal cervical tissue. Consistent with these signatures, IMP3 silencing produced pronounced effects on lipid metabolic readouts in vitro. Together, these data identify IMP3 as a regulator of energy and lipid metabolism in cervical cancer and support its evaluation as a biomarker and potential therapeutic target.

## 1. Introduction

IMP3 (insulin-like growth factor 2 mRNA-binding protein 3; IGF2BP3) is a member of the IGF2BP1/2/3 family that regulates post-transcriptional gene expression by controlling mRNA localization, stability and translation [1,2,3,4]. These KH-domain proteins recognize sequence/structural elements—often within untranslated regions or coding sequences, including at m6A-modified sites—to tune translational output [5]. IMP3 is an oncofetal protein: it is highly expressed during embryogenesis, largely silent in most adult tissues, and re-expressed in diverse cancers^1^. Elevated IMP3 correlates with aggressive phenotypes (proliferation, migration, invasion) and poor clinical outcomes [6]. Nevertheless, its cell-intrinsic functions and direct targets in tumorigenesis remain incompletely defined, with emerging evidence indicating that IMP3 promotes oncogenic programs by stabilizing and enhancing translation of key mRNAs.

Cervical cancer is a leading gynecological malignancy worldwide. According to recent estimates, the worldwide 5-year prevalence stands at 1,948,521 cases, with a rate of 49.8 per 100,000 women in 2022 [7]. Despite being largely preventable through vaccination, screening, and early treatment, the disease accounted for approximately 660,000 new cases and 350,000 deaths in the same year. The etiology is driven by persistent infection with high-risk human papillomaviruses (HR-HPVs), particularly types 16 and 18 [8]. Disease progression reflects integration and expression of the viral E6/E7 oncogenes, functional inactivation of p53 and Rb, genomic instability with stepwise mutation accumulation, and the emergence of precancerous lesions [9]. These events converge on interconnected signaling networks, including the E6/E7–p53/Rb axis [10], PI3K–AKT–mTOR [11], Wnt/β-catenin [12], TGF-β [13], NF-κB [14] and HIF-1α–VEGF pathways [15]. Aberrations across these circuits promote tumor initiation, progression and metastasis, and they constitute active targets for therapeutic development.

Metabolites regulate fundamental cellular processes, including energy production, cell growth and biosynthesis, and adaptation to environmental change [16]. Within the landscape of oncology, these metabolites undergo distinct reconfigurations to fuel the unrestrained proliferation and resilience emblematic of cancerous cells. Such cells reprogramme core metabolic pathways—including glycolysis, the tricarboxylic acid (TCA) cycle, glutaminolysis and lipid metabolism—to sustain rapid proliferation. This pervasive metabolic phenotype—known as the Warburg effect [17]—denotes the propensity of cancer cells to exhibit elevated glucose uptake and lactate production despite sufficient oxygen availability (aerobic glycolysis) [18,19]. Beyond this, metabolites shape energy production, biomass synthesis, redox homeostasis and drug metabolism, and can serve as biomarkers [20,21]. Dissecting these interactions clarifies tumor evolution and progression and reveals therapeutic opportunities.

Here, we test the hypothesis that IMP3 regulates mitochondrial function and lipid metabolic programs in cervical cancer. We first quantified mitochondrial respiration in HeLa cells following RNAi-mediated IMP3 knockdown (IMP3-KD) and assessed complementary indices of cellular energy status (e.g., ATP/ADP and NAD(P)H ratios). To place these phenotypes in a clinical context, we analyzed transcriptomic profiles from normal cervical tissue, primary tumor and paracarcinoma samples to identify differentially expressed genes and pathways, followed by protein–protein interaction network construction and Gene Ontology enrichment to nominate candidate effectors of tumorigenesis. Finally, we profiled metabolites in HeLa cells with and without IMP3-KD and integrated these data with the transcriptomic signatures to connect IMP3-dependent gene regulation to metabolic outputs. Together, this multi-layer framework links IMP3 to coordinated changes in respiration and lipid metabolism, delineates gene networks that accompany these shifts in patient tissues, and highlights metabolite-associated proteins as potential therapeutic entry points in cervical cancer.

## 2. Experimental Methods

### 2.1. Cell Culture

The HeLa (RRID:CVCL_0030), SiHa (RRID:CVCL_0032), and C-33A (RRID:CVCL_1094) cell lines were procured from the American Type Culture Collection (ATCC). Prior to any cell-based experiments, cells were used at a passage number of less than 10. The cellular growth milieu consisted of Dulbecco’s Modified Eagle’s Medium (DMEM) containing 1 g/L glucose, a product of Hyclone. This medium was further enriched with a cocktail of supplements: 10% *v*/*v* fetal bovine serum (Hyclone) to support growth and vitality, 10 U/mL penicillin/streptomycin (Hyclone) to ensure an aseptic environment by countering potential bacterial contamination, and an added 2 mM L-glutamine (Hyclone) to bolster cell metabolism and health. All cells were maintained at 37 °C in a humidified incubator with 5% CO_2_. For all experiments, cells were seeded at 1  ×  10^5^ cells mL^−1^ to ensure a consistent starting density.

### 2.2. Oxygen Consumption Rate (OCR) Examination

OCR was examined using the XF24 Seahorse Metabolic Analyzer from Seahorse Biosciences (North Billerica, MA, USA). Briefly, HeLa cells were plated at a seeding density of 1 × 10^6^ cells/well in 500 μL of complete media in the culture plate. For the mitochondrial stress test (extracellular flux analysis), all inhibitor solutions were prepared according to the manufacturer’s instructions. Subsequently, 1.26 μM oligomycin was injected in port A, 0.67 μM FCCP fluoro-carbonyl cyanide phenylhydrazone in port B, and 0.2 μM rotenone/1 μM antimycin A in port C.

### 2.3. LC–MS/MS Metabolite Profiling

Polar metabolites were quantified by electrospray ionization LC–MS/MS operated in multiple-reaction monitoring (MRM) mode on an Agilent 6410B triple quadrupole mass spectrometer coupled to a 1200 Series HPLC (Agilent Technologies, Santa Clara, CA, USA). Cell pellets were washed, resuspended in 80% methanol, and extracted on ice. Where indicated, 50 μL of mouse peritoneal lavage (4× concentrated) was mixed with 450 μL methanol and processed identically. Extracts were cleared by centrifugation at 4 °C, and the methanol–water supernatant (polar fraction) was collected, dried in a vacuum concentrator, stored at −80 °C, and reconstituted in Milli-Q water on the day of analysis. Reverse-phase separations were performed at 0.4 mL min^−1^ using a Zorbax SB-C18 column (1.8 μm; amino acids, Agilent Technologies) or an Eclipse Plus C18 column (1.8 μm; TCA intermediates, Agilent Technologies). Quantification used external calibration curves prepared at four concentrations and processed in parallel with samples; peak areas were fitted by regression within the linear response range.

### 2.4. ATP/ADP Ratio

ATP and ADP were measured using a luciferin/luciferase bioluminescent assay (ApoSensor ATP:ADP Ratio Assay, Eppendorf, Hamburg, Germany) according to the manufacturer’s instructions. Following PBS wash, cells cultured under high- or low-glucose conditions were detached with trypsin/EDTA and counted; 10,000 cells were assayed per measurement, in duplicate. Luminescence (RLU) was recorded on a Lumat LB9507 luminometer (Berthold Technologies, Bad Wildbad, Germany) and normalized to total protein determined by the Bradford assay (Bio-Rad, Hercules, CA, USA).

### 2.5. NAD^+^/NADH

Total and mitochondrial NAD^+^/NADH were quantified using the Amplite Colorimetric NAD^+^/NADH Assay (AAT Bioquest, Sunnyvale, CA, USA) per the manufacturer’s protocol. Mitochondria were isolated with the Cell Mitochondria Isolation Kit (Beyotime, Shanghai, China) following the supplied instructions to minimize cytosolic contamination.

### 2.6. Public Datasets and Preprocessing

mRNA expression data for IMP3 in cervical carcinoma were obtained from the Gene Expression Omnibus (GEO; accession GSE192804). Data were imported and managed in R/RStudio (R-4.5.0, for Rstudio 2025.09.2+418) using rio (1.2.4) and dplyr (1.1.4).

### 2.7. Differential Expression Analysis

Differentially expressed genes (DEGs) were identified with DESeq2, using its negative binomial framework and Benjamini–Hochberg adjustment for multiple testing. Unless otherwise stated, analytical settings followed package defaults.

### 2.8. Visualization

Volcano plots and heat maps were generated in R with pheatmap. Overlaps among DEG sets were illustrated with FunRich (v3.1.3).

### 2.9. Network Analysis

Protein–protein interaction networks were constructed with STRING (organism set to *Homo sapiens*), and visualized/refined in Cytoscape (v3.6.0).

### 2.10. Functional Enrichment

Functional annotation of DEGs was performed with Metascape (https://metascape.org/ (accessed on 14 January 2023); default parameters) and g:Profiler (https://biit.cs.ut.ee/gprofiler (accessed on 14 January 2023); organism = *Homo sapiens*, “ordered query” and “run as multiquery” enabled). Result tables were downloaded as CSV files for downstream use.

### 2.11. Gene Set Enrichment Analysis

Gene Set Enrichment Analysis (GSEA v4.3.2) was conducted using the Reactome collection from MSigDB (v2025.1.Hs). Default parameters were applied; significance was defined as NOM *p* < 0.05, FDR q < 0.05, and |NES| > 1.0.

### 2.12. Plasmid Construction and Transfections

siRNAs of IMP3 were purchased from GenePharma (Shanghai, China), the target sequence is IMP3-siRNA1: 5′-GCAGGAAUUGACGCUGUAUTT-3′; IMP3-siRNA2: 5′ GCUUCUAUGAAUCUUCAAGTT-3′; Scramble forward: 5′-UUCUCCGAACGUGUCACGUTT -3′.

The human full cDNA sequence of IMP3 was purchased from GenePharma (Shanghai, China). lentiviruses of IMP3-shRNA and non-target control, lentiviruses of IMP3, and empty vector control were also purchased from GenePharma (Shanghai, China). Transfection and virus infection were performed as previously described [22]. The knockdown efficiencies of IMP3 were confirmed by qRT-PCR.

### 2.13. RNA Extraction and cDNA Synthesis

Total RNA was isolated using TRItidy G reagent (PanReac AppliChem, T9424, Darmstadt, Germany) according to the manufacturer’s instructions. RNA quantity and purity were assessed by spectrophotometry (A260/280); integrity was verified by agarose gel electrophoresis. cDNA was synthesized from 1 µg total RNA with the PrimeScript™ RT Reagent Kit (Perfect Real Time; Takara, Shiga, Japan), including the gDNA removal step (gDNA Eraser), following the supplier’s protocol.

### 2.14. RT–qPCR

Quantitative PCR was performed with SYBR^®^ Premix Ex Taq (Tli RNase H Plus; Takara, Beijing, China) on a QuantStudio™ 3 real-time PCR system (Thermo Fisher Scientific, Waltham, MA, USA). Primers were designed and checked with NCBI Primer-BLAST (https://www.ncbi.nlm.nih.gov/tools/primer-blast/ (accessed on 20 October 2025)); amplicons were selected to be specific and, where possible, span exon–exon junctions. Primer sequences: IMP3 forward 5′-ATGACTCCTCCCTACCCG-3′, reverse 5′-GAAAGCTGCTTGATGTGC-3′; GAPDH forward 5′-AGAAGGCTTGGGCTCATTTG-3′, reverse 5′-AGGGGCCATCGACAGTCTTC-3′ (Sigma-Aldrich, Shanghai, China). Reactions were run in technical triplicate; no-template and no-RT controls were included. Melt-curve analysis confirmed single specific products.

### 2.15. Data Analysis

Relative mRNA abundance was calculated by the 2^−ΔΔCt^ method with GAPDH as the endogenous control. For statistical analyses, the unit of replication was independent biological experiments (*n* = 3); technical replicates were averaged within each experiment before inference.

### 2.16. Transmission Electron Microscopy

Cells were processed at 4 °C unless stated otherwise. Suspensions in PBS were pelleted (200× *g*, 5 min) and fixed overnight in Karnovsky’s fixative (2% paraformaldehyde, 2.5% glutaraldehyde in 0.1 M cacodylate buffer, pH 7.4). After three rinses in 0.1 M cacodylate buffer, samples were post-fixed in 1% osmium tetroxide in the same buffer for 1 h, rinsed three times in double-distilled water, and embedded in 2% agar (tryptic soy agar) to form pellets. Agar blocks were trimmed with a Teflon-coated razor and transferred to 50% (*v*/*v*) ethanol for 15 min at room temperature, followed by en bloc staining in uranyl acetate prepared in 70% ethanol for 1 h in the dark. Dehydration was completed through a graded ethanol series (50–100%).

Dehydrated specimens were infiltrated and embedded in epoxy resin and polymerized in a silicone mold. Blocks were inspected under a dissecting microscope, and regions of interest were trimmed. Semi-thin sections (≈0.5 µm) were collected for orientation, and ultrathin sections (75–90 nm) were cut on an Ultracut E (Reichert-Jung, New York, NY, USA) and mounted on Athene thin-bar copper grids (Ted Pella, Redding, CA, USA). Grids were contrasted sequentially with uranyl acetate (in 70% ethanol) and Reynolds’ lead citrate.

Sections were examined on a Hitachi H-300 transmission electron microscope (Chiyoda, Tokyo, Japan). Images were recorded on Kodak EM film 4489 (New York, NY, USA) and developed in Kodak D-19 developer (New York, NY, USA) according to the manufacturer’s instructions. Magnification was verified using a photographic scale marker, and scale bars are provided on all micrographs.

### 2.17. Lipid Metabolism Examination

We performed LC-MS/MS lipidomics on HeLa cell lines post-IMP3 silencing. Lipids were extracted from 1 million cells using Methyl Tert-Butyl Ether (MTBE, Sigma Aldrich, Shanghai, China) and concentrated with a SpeedVac (Thermo Scientific, Waltham, MA, USA). After drying, the lipids were resuspended in a 50% isopropanol and 50% methanol buffer. Analysis was performed on an Ultimate 3000 XRS LC system linked to an Orbitrap Fusion Lumos mass spectrometer (Thermo Scientific). 20 µL lipid solution was loaded onto a 15 cm Accucore Vanquish C18 column (Thermo Fisher Scientific). A specific 28-min LC gradient was used for lipid separation, with defined compositions for mobile phase A and B. The Orbitrap Fusion Lumos, set to both positive and negative ion modes, acquired mass spectra. Parameters for FTMS1 and FTMS2 are specified, with Orbitrap resolutions of 120,000 and 30,000, respectively. Novogene (Tianjin, China) facilitated sub-examination. Data analysis was performed with Rstudio software (https://posit.co/download/rstudio-desktop/ (accessed on 20 October 2025, v2025.09.2+418)), using two-tailed Student’s *t*-test and subsequent Benjamini–Hochberg method for hypothesis correction. The metabolite data was listed in the Supplementary Tables S1 and S2.

### 2.18. Luciferase Reporter Assays

Cell culture and transfection. HeLa cells (ATCC CCL-2; STR authenticated; mycoplasma-negative) were maintained in DMEM with 10% FBS and 1% penicillin/streptomycin at 37 °C (5% CO_2_). For reporter assays, cells were seeded in 24-well plates (1.2 × 10^5^ cells/well) 18–24 h prior to transfection. IMP3 knockdown was achieved using lentiviral shRNA (shIMP3) or non-targeting control (shNT); experiments were performed 72–96 h post-transduction. Where indicated, RNAi-resistant IMP3 (IMP3^res^) or SREBF1 (SREBP1c) ORF plasmids were co-transfected for rescue.

Plasmids. The SREBF1 3′UTR reporter was generated by cloning the full-length human SREBF1 3′UTR downstream of Firefly luciferase (pGL3-Promoter). A mutant 3′UTR (Mut_3UTR) was engineered by substituting CA-rich/KH-domain binding motifs to disrupt IGF2BP3/IMP3 recognition without altering UTR length. The SRE-luc plasmid contained multimerized sterol response elements upstream of a minimal promoter driving Firefly luciferase. Renilla luciferase (pRL-TK) was co-transfected for normalization. IMP3^res carried silent mutations within the shRNA target sequence; SREBP1c was cloned as a mature nSREBP1c ORF where specified.

Transfection and assay. Plasmids were transfected using Lipofectamine 3000 (Thermo Fisher, Waltham, MA, USA) per the manufacturer’s instructions. Per well: Firefly reporter 200 ng, Renilla 20 ng, ± IMP3^res^ or SREBP1c ORF 200 ng, topped to equal total DNA with empty vector. Cells were lysed 24–36 h post-transfection; luciferase activity was quantified using a dual-luciferase kit (Promega, Madison, WI, USA) on a luminometer with 10 s integration time. Firefly signals were normalized to Renilla (Firefly/Renilla).

### 2.19. Enzymatic Activity Assays

Cell treatment. HeLa cells were transduced with non-targeting control (shNT), shIMP3, or shIMP3 + RNAi-resistant IMP3 (IMP3^res^). Assays were performed 72–96 h post-transduction. Cells were harvested on ice; all steps used pre-chilled buffers.

Lysate preparation. Cells were washed (PBS, ice-cold) and lysed in enzyme assay buffer (50 mM Tris-HCl, pH 7.4, 150 mM NaCl, 1 mM EDTA, 1 mM DTT, 0.1% Triton X-100) supplemented with protease inhibitors. Lysates were clarified (12,000× *g*, 10 min, 4 °C); protein was quantified by BCA.

IDH1 (NADP^+^-dependent) activity. Reactions (200 µL) contained 50 mM Tris-HCl, pH 7.4, 1 mM NADP^+^, 2 mM MgCl_2_, 2 mM D,L-isocitrate, and 10–20 µg lysate protein. NADPH production was monitored at A_340_ nm (ε = 6220 M^−1^ cm^−1^) at 30 °C for 5–10 min; the initial linear rate was used to calculate nmol NADPH·min^−1^·mg^−1^. For specificity, parallel reactions without substrate or without NADP^+^ served as blanks; where indicated, (R)-2-hydroxyglutarate or IDH inhibitors can be excluded to avoid confounds.

IDH2 (NADP^+^) and IDH3 (NAD^+^). As above, substituting mitochondrial-enriched fraction (digitonin-permeabilization or differential centrifugation), and using NADP^+^ + isocitrate for IDH2 or NAD^+^ + isocitrate + Mn^2+^/Mg^2+^ for IDH3. Rates were normalized to protein.

Fumarase activity. Monitored by the decrease in A_240_ nm as fumarate to malate (ε_fumarate ≈ 2.44 mM^−1^ cm^−1^) in 50 mM potassium phosphate buffer (pH 7.4) at 30 °C, with 10–20 µg lysate.

MDH2 activity. NADH oxidation at A_340_ nm upon addition of oxaloacetate (0.2–0.5 mM) in 50 mM Tris-HCl, pH 7.4, 1 mM EDTA, 0.2 mM NADH.

Citrate synthase (CS). Standard DTNB-coupled assay: reaction mix with acetyl-CoA (0.1 mM), oxaloacetate (0.5 mM), and DTNB (0.1 mM); TNB formation at A_412_ nm measured as a mitochondrial mass/quality control.

Normalization and QC. Activities were blank-corrected and normalized to protein; linearity was verified with time and protein input. Technical duplicates were averaged within each biological replicate.

### 2.20. Statistical Analysis

All experiments were performed as independent biological replicates on separate days (minimum *n* = 3). Technical replicates were used only to estimate within-experiment variability and were averaged before between-experiment analyses. Unless stated otherwise, quantitative data are reported as mean ± s.d., with *n* denoting the number of independent experiments. Representative images in figures were selected from ≥3 independent experiments and reflect the aggregate quantitative results.

Comparisons between two groups used two-sided tests (Student’s *t*-test; Welch’s correction applied when variances were unequal). Categorical variables were analyzed by χ^2^ or Fisher’s exact test, as appropriate. Survival was evaluated using Cox proportional hazards models (univariate and multivariable), reporting hazard ratios (HRs) with 95% confidence intervals; proportional-hazards assumptions were assessed. Where multiple comparisons were performed, *p* values were adjusted using the Benjamini–Hochberg false discovery rate (FDR) procedure, with significance defined as FDR-adjusted *q* < 0.05 (or two-sided *p* < 0.05 when a single hypothesis was tested). Analyses were conducted in SPSS v20.0 (IBM).

## 3. Results

### 3.1. Silencing of IMP3 Alters HeLa Cell Mitochondrial Function

**Figure 1:**
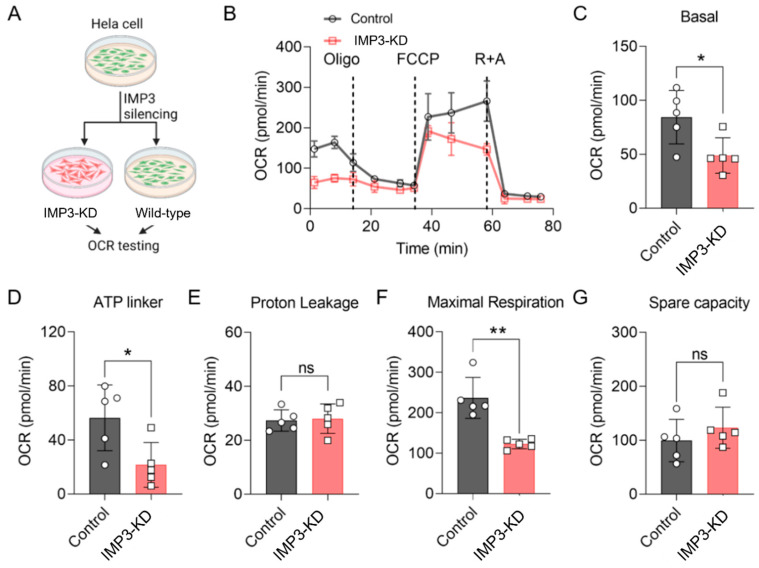
**IMP3 down-regulation modulates the oxygen consumption rate (OCR) in HeLa cells**. (**A**). Diagram of the OCR assay procedure. (**B**). Comparative OCR in HeLa cells following IMP3 down-regulation, assessed via Seahorse (*n* = 5 replicates per group). Cells underwent treatment with oligomycin A, carbonyl cyanide-p-trifluoromethoxyphenylhydrazon (FCCP), rotenone and antimycin A. (**C**–**G**). Distinct OCR parameters delineate mitochondrial respiratory states: Basal respiration (**C**); ATP-linked respiration (**D**); Proton leak (**E**); Maximal respiration (**F**); Spare respiratory capacity (**G**). N = 5 replicates per group. Data were represented as mean ± SEM; ns, no significance, * *p* < 0.05, ** *p* < 0.01. Statistical results were obtained by employing unpaired Student’s *t*-test.

Previous studies have highlighted the pivotal role of mitochondrial metabolism in regulating cellular energy dynamics, particularly in human cervical cancer cell lines such as HeLa2 [23,24]. In the present study, we investigated the impact of IMP3 downregulation on energy metabolic pathways in HeLa cells. As shown in Figure 1A, IMP3 expression was effectively reduced using targeted shRNA approaches (Supplementary Figure S1 and Table 1). The oxygen consumption rate (OCR) of these cells was subsequently assessed using the Seahorse analytic platform. IMP3 knockdown (IMP3-KD) HeLa cells exhibited a marked decrease in OCR compared with control cells (Figure 1B). Further analysis revealed that IMP3 silencing significantly reduced key bioenergetic parameters, including basal respiration (Figure 1C), ATP-linked respiration (Figure 1D), and maximal respiration (Figure 1F). By contrast, proton leak (Figure 1E) and metabolic spare capacity (Figure 1G) were unaffected. Together, these results demonstrate that IMP3 downregulation exerts a pronounced effect on the mitochondrial function of HeLa cells, predominantly through the suppression of mitochondrial respiratory function.

### 3.2. Silencing of IMP3 Alters the Energy Metabolism

**Figure 2:**
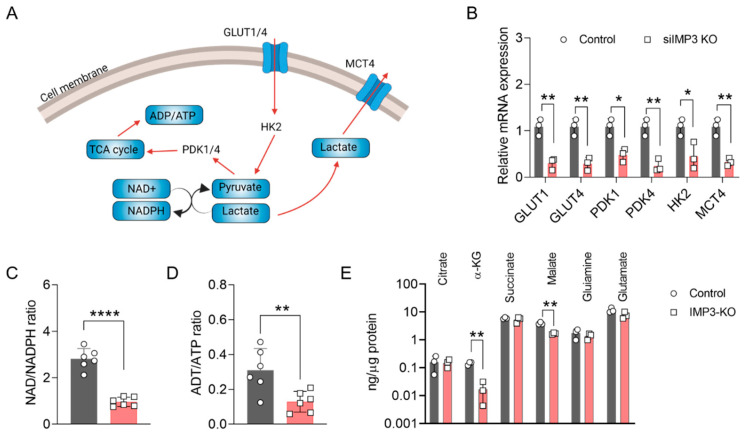
**Altered Energy Metabolism in HeLa Cells Following IMP3 Down-regulation.** (**A**) Conceptual representation detailing the role of ATP and NADPH in cellular energy metabolism processes. Red curve is metabolism flow direction, and grey curve is the translate direction. (**B**) Comparative analysis of the relative mRNA expressions of the specified genes against IMP3-KD Hela cells, conducted over *n* = 3 replicates. (**C**) Examination of the NAD-NADPH ratio differentials observed between wild-type and IMP3-KD Hela phenotypes, sampled across *n* = 6 replicates. (**D**) Contrast in the ADP-ATP ratios for wild-type and IMP3-KD Hela cell lines, derived from *n* = 6 replicates. (**E**) Comprehensive quantification of metabolite concentrations distinguishing the wild-type from the IMP3-KD Hela cells. Data were represented as mean ± SEM; ns, no significance, * *p* < 0.05, ** *p* < 0.01, **** *p* < 0.0001. Statistical results were obtained by employing unpaired Student’s *t*-test.

Within the complex network of cellular energy metabolism, glucose metabolism plays a central role in sustaining energy production essential for cellular function. Key intermediates in this process—pyruvate and lactate—are integral to the glycolytic pathway (Figure 2A). To explore the impact of IMP3 loss on glycolytic regulation, we first examined the transcriptional profile of genes involved in pyruvate and lactate metabolism. As shown in Figure 2B, several pivotal genes, including *GLUT1*, *GLUT3*, *PDK1*, *PDK4*, *HK2*, and *MCT4*, were markedly downregulated in IMP3-knockdown (IMP3-KD) HeLa cells compared with controls. We next assessed canonical indicators of cellular energy metabolism by quantifying the ADP/ATP and NAD^+^/NADPH ratios following IMP3 silencing. As depicted in Figure 2C,D, both ratios were significantly reduced relative to control cells. These findings suggest that IMP3 depletion disrupts glycolytic and mitochondrial metabolic capacity, thereby impairing the bioenergetic efficiency of HeLa cells.

To further delineate the effects of IMP3 silencing on mitochondrial metabolism, we performed comprehensive metabolite profiling. As shown in Figure 2E, the levels of α-ketoglutarate (α-KG) and malate were markedly reduced in IMP3- knockdown HeLa cells. The production of α-KG and malate is catalyzed primarily by isocitrate dehydrogenase 1 (IDH1) and fumarase, respectively, with IDH1 also contributing to the regulation of the NAD+/NADPH balance. These findings implicate IDH1 as a potential key mediator in the metabolic cascade initiated by IMP3 depletion. Future studies should aim to dissect the functional interplay between IMP3 and IDH1, thereby elucidating its role in mitochondrial metabolic regulation and its broader implications for cervical cancer initiation and progression.

**Figure 3:**
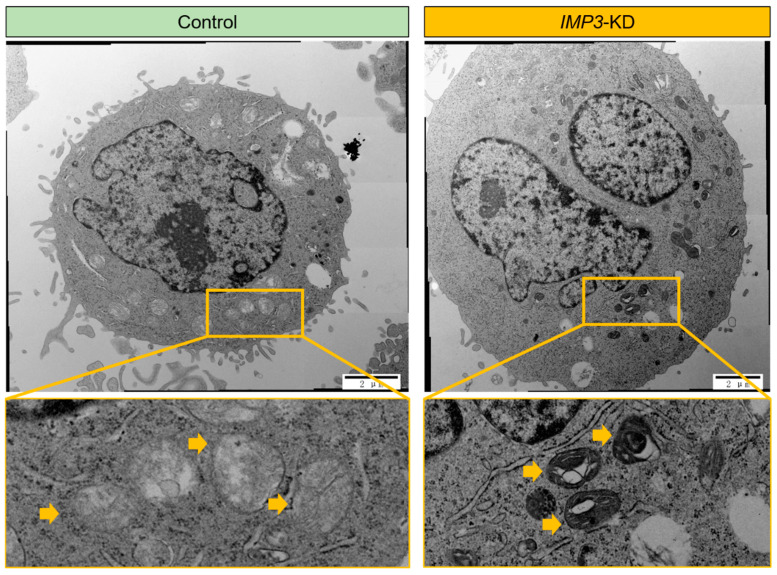
**IMP3 depletion induces pronounced ultrastructural changes in autophagic compartments.** Transmission electron micrographs of HeLa under Control (**left**) and IMP3-KD (**right**) conditions. In control cells, discrete, double-membrane autophagosomes (yellow arrowheads) with relatively uniform electron-dense contents are observed (**top left**), and higher-magnification insets (**bottom left**) show compact, spherical vesicles. By contrast, IMP3-depleted cells accumulate numerous, enlarged autophagic vacuoles with multi-lamellar, concentric membrane whorls (yellow arrowheads; **top right**), as highlighted in the high-power inset (**bottom right**). These data indicate that loss of IMP3 disrupts autophagosome maturation and leads to the build-up of aberrant, multi-lamellar structures. Scale bars: 2 µm (**top panels**) and 500 nm (**bottom panels**).

To further investigate the impact of IMP3 depletion, we employed high-resolution transmission electron microscopy (TEM) to examine the ultrastructure of HeLa cells. As shown in Figure 3, silencing IMP3 did not markedly affect the overall morphology of most cellular organelles. However, mitochondria represented a clear exception. Detailed TEM analyses revealed pronounced structural abnormalities in IMP3- knockdown cells, including disrupted cristae organization and occasional outer membrane rupture. These ultrastructural alterations are characteristic of impaired mitochondrial integrity and function, supporting the notion that IMP3 loss compromises both the architecture and bioenergetic competence of mitochondria.

### 3.3. Bioinformatics Analysis of Overexpressed IMP3 in Vertical Cancer Tumorigenesis

**Figure 4:**
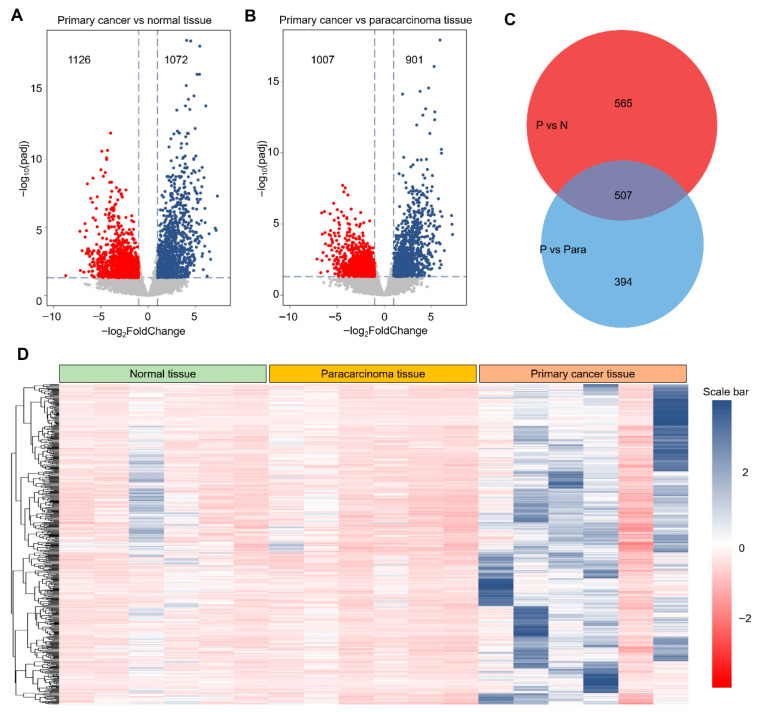
**Identification of differentially expressed genes from the GEO database.** (**A**). Representative volcano plot of gene expression in primary cancer tissue compared with normal cervical tissue. Red dot presented as the down-regulation DEGs, and blue dot presented as the up-regulated DEGs. (**B**). Representative volcano plot of gene expression in primary cancer tissue compared with paracarcinoma tissue. (**C**). Venn diagram of up-regulated DEGs obtained from (**A**,**B**). (**D**). Representative heatmap of identified 507 DEGs from normal tissue to primary cancer tissue. Threshold value to consider as significance is |log2FoldChange| > 1.0 and padj < 0.05. Red dots and blue dots are contributed as down-regulated and up-regulated DEGs, respectively. The value of gene expression was normalized using the Z-score method in the row direction.

Derived from GEO datasets, the constructed database was stratified into three distinct categories: healthy cervical tissue, paracarcinoma tissue, and primary cervical cancer tissue, providing a comprehensive view of the tumorigenic continuum in cervical cancer and enabling multi-omics analyses to identify key regulatory genes. Using gene expression profiles obtained from the GEO database, we performed differential expression analyses comparing primary cancer tissues with both healthy and paracarcinoma tissues. As shown in Figure 4A,B, these comparisons identified 2198 and 1908 differentially expressed genes (DEGs), respectively. We next focused on the subset of upregulated DEGs, hypothesizing that these genes are more likely to exhibit proto-oncogenic properties. A Venn diagram (Figure 4C) revealed an intersection of 507 upregulated DEGs shared between the two comparisons. These candidates may exert significant influence on cervical tumorigenesis. To further characterize this subset, we visualized their expression patterns using a heatmap (Figure 4D), which revealed a consistent and marked upregulation relative to both healthy and paracarcinoma tissues. Collectively, these findings suggest that this curated gene set is closely associated with the molecular reprogramming events underlying cervical cancer initiation and progression.

**Figure 5:**
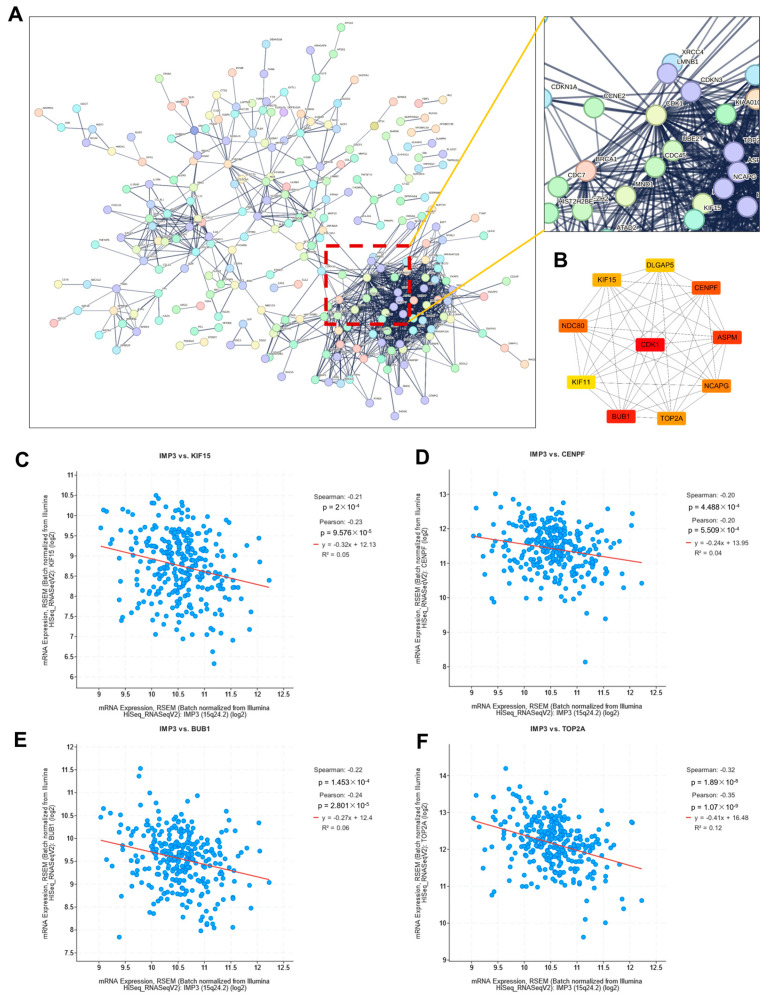
**Identification of critical hub genes associated with *IMP3* expression in cervical cancer.** (**A**). Protein–protein interaction network of identified DEGs. Network was obtained from the STRING database. An extended image displayed the CDK1 interaction network. (**B**). Protein–protein interaction network of top-10 hub genes. (**C**–**F**). Correlation images of IMP3 and identified hub genes. The threshold value of correlation to consider as significance is *p* < 0.05.

To investigate how these DEGs participate in regulatory networks driving tumorigenesis, we performed protein–protein interaction (PPI) analysis. As shown in Figure 5A, a high-confidence PPI network (confidence score > 0.900) was constructed using the STRING database, highlighting key gene–gene interactions. The resulting network was segregated into discrete clusters, with one predominant cluster enriched for a large proportion of DEGs, as illustrated in the magnified view in Figure 5A. Notably, this cluster was organized around a central hub defined by CDK1.

Recent investigations, such as those by Zhu et al. [25], unveiled CDK1 as a linchpin connecting NF-κB and β-catenin signaling pathways in gastric tumorigenesis. In a parallel revelation, Zhong et al. [26] highlighted KCTD12’s role in propelling tumorigenesis, modulated through a CDC25B/CDK1/Aurora-A dependent G2/M transition. Such findings foster the hypothesis that CDK1 might be instrumental in tumorigenesis, either by modulating cell cycle transitions or through activating inflammation-associated pathways.

To further refine our network analysis, we employed the Cytoscape (3.10.4) Hubba plugin to identify the top 10 hub genes. Notably, most of these genes can be classified as transcription factors. As illustrated in Figure 5B, CDK1 occupied a central position within the PPI network. Consistent with previous reports, IMP3 has been shown to cooperate with circCDKN2B-AS1 to promote aerobic glycolysis in cervical squamous cell carcinoma [27]. To assess potential associations between IMP3 and the identified hub genes, we performed correlation analysis, which revealed significant relationships with several candidates, including KIF15, CENPF, BUB1, and TOP2A (Figure 5C). Interestingly, these associations were predominantly negative, indicating that higher IMP3 expression is linked to lower expression of these hub genes in cervical cancer.

The inverse associations between IMP3 and a subset of mitotic spindle/kinetochore hub genes (e.g., KIF15, CENPF, TOP2A; BUB1, showing a distinct behavior) suggest reciprocal coupling between a lipogenic program and G2/M circuitry [28,29]. In line with this, IMP3 silencing diminishes mitochondrial respiration [30] and SREBP-driven lipogenesis [31], yet elevates several mitotic transcripts, consistent with a compensatory G2/M response under reduced lipid/energy supply. We therefore propose that IMP3 biases cervical cancer cells toward a lipid-addicted state, whereas IMP3 loss shifts cells toward a mitotic-checkpoint–enriched state operating under metabolic constraint.

**Figure 6:**
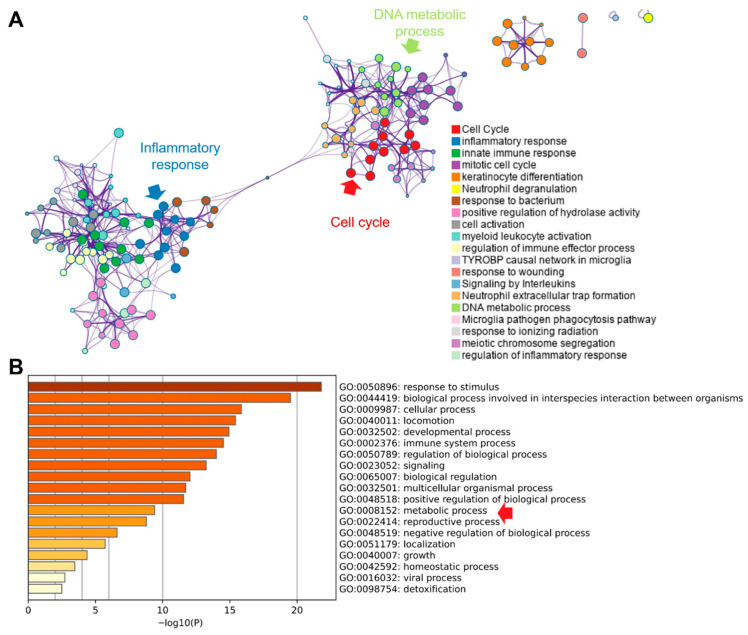
**Pathway enrichment of identified DEGs from the Metascape database**. (**A**). Enriched pathway network analysis. (**B**). Top-20 enriched GO terms. Red raw indicted the critical pathway.

Pathway enrichment of differentially expressed genes using Metascape resolved two principal modules (Figure 6A): (i) inflammatory and innate immune responses and (ii) cell-cycle control. “Extracellular trap formation” bridged these modules, implicating neutrophil extracellular traps (NETs) in linking immune activation with proliferative programs [32]. Consistent with this, GO terms related to DNA metabolism—encompassing replication and repair—were enriched, aligning with the increased demand for nucleotide synthesis during cell-cycle progression. Additional GO analysis (top 20 terms) was dominated by inflammation- and cell-process categories, with inclusion of the broad metabolic process (GO:0008152), which subsumes anabolic and catabolic pathways that supply precursors for macromolecular biosynthesis. These enrichment patterns indicate that tumor gene-expression changes converge on coordinated activation of inflammatory signaling and cell-cycle progression, with NET-associated biology potentially coupling these axes. The prominence of DNA metabolic processes and the overarching metabolic process term is consistent with a reinforced metabolic infrastructure that supports sustained proliferation, biomass accumulation and genome duplication—hallmarks of tumorigenesis.

### 3.4. Silencing of IMP3 Alters Lipid Metabolism of HeLa Cells

**Figure 7:**
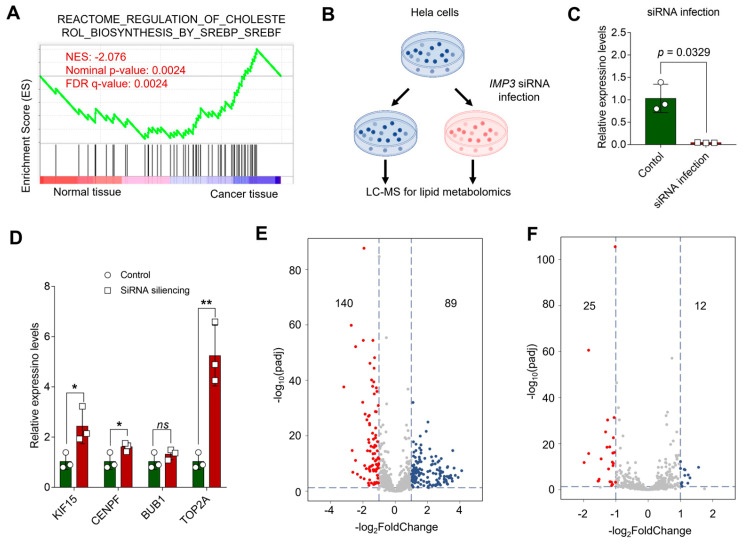
**Unraveling the Interplay between Cholesterol Pathways and IMP3 Silencing in HeLa Cells**. (**A**). GSEA profiling juxtaposes cholesterol-associated pathways between normal and cancer tissues, with the emphasis on the Normalized Enrichment Score (NES). (**B**). Conceptual schema delineating the cascade of events post-IMP3 silencing in HeLa cells, culminating in a lipid metabolomics assessment. (**C**). Quantitative delineation of IMP3 expression disparities between control and siRNA-infected cohorts. N = 3 replicates per group. Statistical validity affirmed using Student’s *t*-test, where *p*-values less than 0.05 denote statistical significance. (**D**). Comparative expression metrics for the pivotal hub genes following IMP3 suppression, juxtaposed with the control ensemble. Each assessment entails three replicates. N = 3 replicates per group. Statistical demarcation via Student’s *t*-test: *, *p* < 0.05; **, *p* < 0.01; “ns” signifies lack of statistical distinction. (**E**,**F**). Exemplar volcano plots elucidating lipid metabolite spectra in Hela cells post-IMP3-KD, vis-a-vis the control group. (**E**) reflects the positive ion mode, while (**F**) captures the negative ion mode. Multiple-testing: BH-FDR. The threshold demarcating significance is steered by criteria: |log_2_FoldChange| > 1.0 & padj < 0.05.

Lipid metabolic pathways have emerged as critical determinants of tumor growth and therapeutic response [33,34]. Gene set enrichment analysis (GSEA) of GEO-derived expression profiles indicated that cervical cancer tumorigenesis is closely linked to SREBF/SREBP-driven biosynthetic programs, highlighting cholesterol biosynthesis (Figure 7A). To test whether IMP3 modulates cholesterol-centric metabolism, we generated HeLa cells with shRNA-mediated IMP3 knockdown (Figure 7B and Figure S2) and confirmed efficient silencing by RT–qPCR (Figure 7C). Consistent with our PPI-based hub-gene analysis, RT–qPCR showed that most prioritized genes—including KIF15, CENPF, and TOP2A—were upregulated following IMP3 depletion, with BUB1 as an exception (Figure 7D). Additionally, we also validated in the SiHa and C-33A cells lines, and presented similar results (Supplementary Figure S8).

Lipidomic profiling further revealed broad remodeling of the lipidome relative to controls: volcano plots summarizing positive and negative ion modes (Figure 7E,F) identified 140 decreased and 89 increased metabolites in positive mode, and 25 decreased and 12 increased in negative mode under stringent significance criteria. SREBP1c is a master regulator of de novo lipogenesis, upregulating enzymes such as FASN and ACC1 [35]. Prior studies in hepatocellular and breast cancers show that IGF2BP3 (IMP3) binds the 3′ UTR of SREBP1c mRNA, stabilizing the transcript and enhancing SREBP1c nuclear accumulation and transactivation of lipogenic targets, including FASN [36,37]. In line with these reports, IMP3 knockdown markedly reduced key lipid species—triacylglycerols, diacylglycerols, phospholipids, and cholesterol derivatives (Figure 7E,F)—consistent with attenuated SREBP1c/FASN activity. Together, these findings support a model in which IMP3 functions upstream of the SREBP–FASN axis as an mRNA-binding amplifier of lipogenic signaling, promoting lipid accumulation in cervical cancer cells and suggesting a tractable metabolic vulnerability.

Alterations in lipid composition influence core cellular processes and functions [38]. In our lipidomic analysis, long-chain acylcarnitines (e.g., O-oleoylcarnitine)—intermediates that shuttle fatty acyl groups into mitochondria for β-oxidation—were significantly depleted following IMP3 silencing, indicating reduced mitochondrial fatty-acid flux. Hydroxylated acylcarnitines, including 3-hydroxyoctadecanoylcarnitine and 3-hydroxybutyrylcarnitine, were likewise diminished, consistent with a blockade of β-oxidation and impaired buffering of acyl-CoA pools. Polyunsaturated phosphatidylcholines (PCs), such as PC(20:2/22:6) and ether-linked PC(22:5e/13:0), which enhance membrane fluidity and serve as precursors for lipid mediators [39], were downregulated, suggesting disrupted bilayer dynamics and reduced availability of signaling lipids. By contrast, the unusual short-chain symmetric PC(9:0/9:0) was increased, consistent with compensatory membrane remodeling that may alter curvature and promote non-lamellar domain formation. Notably, the sphingomyelin species SM(d15:1/19:0)—enriched in lipid rafts that scaffold receptor signaling [40]—accumulated upon IMP3 knockdown, pointing to sphingolipid-driven reorganization of membrane microdomains and potential shifts in pro-survival signaling. Together, these coordinated changes indicate that IMP3 silencing not only constrains fatty-acid catabolism but also rewires membrane lipid composition, with likely consequences for cellular energetics, signaling, and structural integrity.

**Figure 8:**
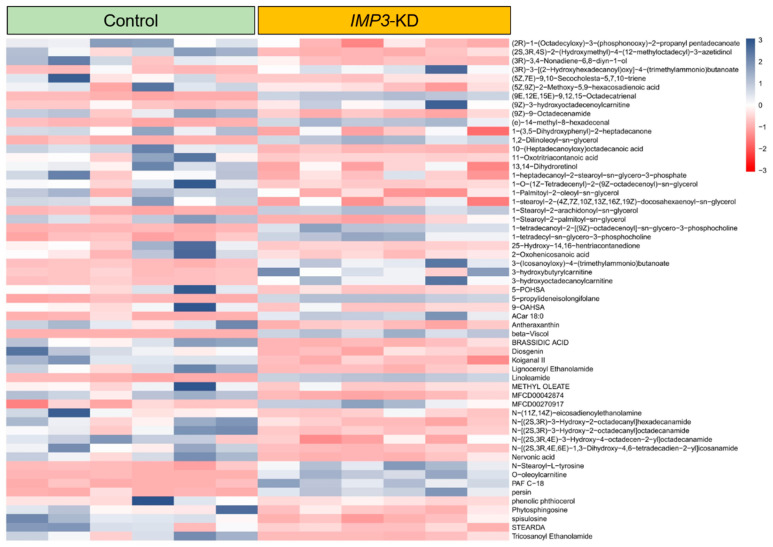
**Differential metabolite expression heatmap in IMP3-KD Hela cells using standard compounds.** Individual rows correspond to distinct metabolites, while columns signify separate experimental batches. The color gradient, based on z-scores, showcases metabolite abundance: blue denotes elevated levels, whereas red indicates reduced concentrations.

In our metabolite survey of HeLa cells, Figure 8 and Figures S3–S7 present differentially expressed metabolites as heatmaps. Most altered species fall within long-chain lipid classes, with full annotations provided in Supplementary Information Files S1–S4. Analyses were stratified by ionization mode; in negative ion mode, the heatmaps highlight pronounced shifts in Cer-NDS/NS, cardiolipin (CL), and diacylglycerols (DG/DAG) following IMP3 shRNA–mediated knockdown.

Within the Cer-NDS class, several long-chain N-acyl species—including Cer-NDS (d18:0/18:0), (d18:0/22:0), (d18:0/23:0), and (d18:0/24:0)—displayed marked changes (Supplementary Figure S1). Given their role as core membrane constituents, alterations in these ceramides, together with shifts in CL and DG, suggest that IMP3 silencing drives substantive remodeling of membrane lipid composition, with potential consequences for membrane architecture and function.

In the phosphatidylethanolamine (PE) and triacylglycerol (TAG) classes, IMP3 silencing led to marked downregulation (Supplementary Figures S4 and S6). Within lysophosphatidylcholines (LPC) and phosphatidylcholines (PC), five and seventy species, respectively, showed significant differential abundance in IMP3-deficient versus control cells (Supplementary Figure S3). In the sphingomyelin (SM) class, five metabolites were upregulated and four were downregulated (Supplementary Figure S7). Together, these results underscore the broad impact of IMP3 depletion on lipid metabolic networks.

Class-level inspection revealed a coordinated depletion of TAG/DAG and of PUFA-rich PC/PE species, alongside altered LPC remodeling and selective SM accumulation; CL species were also perturbed. Long-chain and 3-hydroxy acylcarnitines declined, indicative of reduced β-oxidation. These signatures, together with reduced SREBF1/FASN/ACACA transcripts and SRE-luc activity, indicate blunted de novo lipogenesis and membrane reorganization—including mitochondrial inner-membrane remodeling—upon IMP3 knockdown.

Multiple lines of evidence support a clinically relevant role for IMP3 in cervical cancer. Lu et al. [41] reported that IMP3 immunoreactivity is virtually absent in normal cervical epithelium and low-grade intraepithelial lesions but robust in high-grade cervical intraepithelial neoplasia and invasive carcinoma, where it predicts progression. Consistent with this trend, analysis of the TCGA-CESC cohort shows marked upregulation of IGF2BP3 in tumors relative to normal tissue; patients in the highest quartile of IGF2BP3 expression exhibit a tendency toward shorter overall and progression-free survival than those in the lowest quartile, although these differences do not uniformly reach statistical significance. We note that our mechanistic studies were performed in HeLa cells; validation in primary cervical cancer cells and patient-derived models will be essential to generalize IMP3’s impact on lipid metabolism and clinical outcome. Our mechanistic and lipidomic analyses were conducted in HeLa, and thus may not capture the full heterogeneity of cervical cancer across histologic subtypes and HPV backgrounds. Consequently, extrapolation beyond HeLa should be made with caution.

### 3.5. IMP3 Enhances SREBF1 mRNA Output to Promote Lipogenic Transcription

In HeLa cells, an SREBF1 3′UTR Firefly/Renilla reporter showed reduced activity upon IMP3 knockdown (shIMP3) versus shNT (Supplementary Figure S9a), which was restored by RNAi-resistant IMP3. By contrast, a motif-disrupted 3′UTR reporter was insensitive to IMP3 perturbation (no significant differences among shNT/shIMP3/rescue), indicating 3′UTR-dependent regulation of SREBF1 by IMP3. Consistent with this mechanism, sterol response element–driven transcription (SRE-luc) decreased with IMP3 knockdown (Supplementary Figure S9b). Ectopic SREBP1c partially rescued SRE-luc in the knockdown background (~1.10 folds) and increased activity in control cells (~1.20 folds), supporting a functional IMP3–SREBP1c axis linking post-transcriptional control to lipogenic transcription.

### 3.6. IMP3 Knockdown Constrains TCA Flux Through Enzyme-Level Defects at IDH1 and Fumarase

As given in Supplementary Figure S10, targeted enzyme assays in HeLa revealed a marked reduction in IDH1 (NADP^+^-dependent) activity upon IMP3 knockdown, with partial restoration by IMP3 re-expression. Fumarase activity was likewise decreased and partially rescued. By contrast, IDH3 (NAD^+^-dependent), IDH2 (NADP^+^-dependent) and MDH2 activities were minimally affected (not significant), and citrate synthase—a proxy for mitochondrial content—remained unchanged. Immunoblotting showed a modest reduction in IDH1 and fumarase protein abundance (∼0.8–0.85× of shNT), whereas IDH2/IDH3A/MDH2/citrate synthase levels were comparable across groups. The disproportionately larger drop in IDH1 activity relative to its protein abundance, together with reduced α-ketoglutarate and malate levels, indicates that IMP3 loss limits TCA flux predominantly via enzyme-level control at IDH1 and fumarase, rather than via bulk changes in mitochondrial mass.

## 4. Discussion

This study links the oncofetal RNA-binding protein IMP3 (IGF2BP3) to coordinated control of mitochondrial function and lipid metabolism in cervical cancer models, integrating cell-intrinsic physiology with transcriptomic and lipidomic readouts. RNAi-mediated IMP3 depletion in HeLa cells reduced oxygen consumption and ATP-linked respiration without materially altering spare capacity, consistent with a primary defect in mitochondrial energy conversion rather than a global loss of respiratory reserve (Figure 1). Ultrastructural changes—cristae disorganization and occasional outer-membrane rupture—further support compromised mitochondrial integrity (Figure 3). Together with shifts in cellular energy indices (ATP/ADP and pyridine-nucleotide ratios), these data identify IMP3 as a determinant of mitochondrial performance in this context.

Mechanistically, multiple layers of evidence point to node-specific constraints within central carbon metabolism rather than a uniform depression of mitochondrial content. Targeted metabolite profiling showed reduced α-ketoglutarate and malate in *IMP3*-deficient cells, and enzyme assays revealed disproportionate decreases in IDH1 (NADP^+^-dependent) and fumarase activities, with minimal changes in citrate synthase (a proxy for mitochondrial mass) and in IDH2/IDH3/MDH2 (Supplementary Figure S10). The greater loss of activity than abundance for IDH1, together with the observed metabolite deficits, suggests that *IMP3* loss constrains tricarboxylic-acid (TCA) flux at specific enzymatic steps, potentially through post-transcriptional control of metabolic enzymes or cofactors. These findings rationalize the respiration phenotype and provide biochemical entry points for future work, including isotope-tracing to quantify flux redistribution.

*IMP3* knockdown also remodeled the lipidome in a direction consistent with blunted de novo lipogenesis and altered membrane architecture. Lipidomic analyses (BH-FDR controlled) identified coordinated depletion of tri- and di-acylglycerols and PUFA-rich PC/PE species, reductions in long-chain and 3-hydroxy acylcarnitines (suggesting curtailed β-oxidation), and selective alterations in sphingomyelins and cardiolipins that are expected to influence raft organization and inner-mitochondrial membrane function (Figure 7E,F, Figure 8 and Figures S3–S7). At the regulatory level, *IMP3* reduced an SREBF1 3′UTR reporter in a motif-dependent manner and diminished sterol-response–element (SRE) transcriptional activity, with restoration by RNAi-resistant *IMP3* and partial rescue by SREBP1c overexpression (Supplementary Figure S9). These orthogonal readouts support a model in which *IMP3* sustains lipogenic transcription by enhancing SREBF1 mRNA output, thereby promoting lipid accumulation and membrane remodeling in cervical cancer cells.

Patient-anchored transcriptomics places these cell-intrinsic findings in a disease context. Across normal, paracarcinoma and primary tumor specimens, we identified broad gene-expression changes that segregated into two principal modules—innate/inflammatory programs and cell-cycle regulation—with “extracellular trap formation” bridging the two (Metascape; Figure 6). Gene-set analyses highlighted metabolic annotations, including DNA metabolism, in keeping with the biosynthetic demands of proliferating tumor cells (Figure 4, Figure 5 and Figure 6). Within the PPI network, CDK1 emerged as a central hub among up-regulated genes, and several mitotic/kinetochore genes (e.g., KIF15, CENPF, TOP2A) showed significant relationships with IMP3 expression in tumors. Together with our cellular data, these patterns suggest that IMP3 supports an anabolic state coupling lipogenic output and proliferation, whereas IMP3 loss shifts cells towards a mitotic-checkpoint–enriched program operating under metabolic constraint.

The convergence of mitochondrial, lipidomic and transcriptomic signatures has several implications. First, RNA-binding proteins such as *IMP3* can operate as post-transcriptional amplifiers of metabolic programs, aligning translational control with bioenergetic needs. Here, mitochondrial respiration defects co-occurred with reduced SREBF1-dependent activity and lipid-class remodeling, a combination expected to limit membrane biogenesis and signaling lipids while altering organellar physiology. Second, the observed enzyme-level bottlenecks (IDH1, fumarase) nominate discrete metabolic nodes that may be exploitable; for example, reduced NADPH-generating capacity via IDH1 could sensitize cells to redox stress or impair fatty-acid synthesis. Third, the lipid-class shifts (TAG/DAG, PUFA-rich PC/PE, SM/CL) point to altered membrane fluidity, raft composition and mitochondrial inner-membrane dynamics—variables that influence receptor signaling, apoptosis susceptibility and respiratory supercomplex organization.

Our study has limitations. Most mechanistic experiments were conducted in HeLa cells; while we observed concordant transcriptional validations in SiHa and C-33A lines (Supplementary Figure S8), broader generalization will require primary cultures, organoids and in vivo models representing diverse HPV backgrounds and histologies. The RNA-seq analyses provide associative links between *IMP3* expression and pathway activity in patient samples; causal inference awaits perturbation studies in clinical models. Although our lipidomics used independent-experiment replication with BH-FDR control, orthogonal flux measurements (e.g., ^13^C tracing of de novo lipogenesis and β-oxidation, malonyl-CoA quantification) will strengthen the interpretation of pathway directionality. Finally, we deliberately tempered claims about specific phosphorylation events within the lipogenic machinery and instead anchored conclusions on transcriptional reporters, enzyme activities and lipid-class outcomes; targeted phospho-proteomics could refine this axis in future revisions.

## 5. Conclusions

Our comprehensive study delves deeply into the multifaceted role of *IMP3* in orchestrating mitochondrial functionality, energy homeostasis, and lipid metabolic pathways. Concurrently, we employed advanced bioinformatics techniques to discern the potential implications of *IMP3* overexpression in cervical cancer progression. Post-transfection analyses in Hela cells illuminated that a diminished expression of *IMP3* can considerably perturb mitochondrial dynamics and operations. This inference is further corroborated by alterations observed in energy metabolism-centric genes and markers, all of which align with our TEM observations. Leveraging publicly available datasets from the GEO database, our bioinformatics exploration unveiled a marked overexpression of *IMP3* in primary cancer tissues relative to benign cervical tissues. Intriguingly, this upsurge in *IMP3* might be intricately intertwined with the modulation of metabolic pathways, cell cycle progression, and inflammatory responses, all converging to potentially foster the tumorigenesis trajectory in cervical cancer. The culmination of our lipid metabolic investigations shed light on how *IMP3* silencing can substantially drive lipid biosynthesis in vivo. Collectively, our findings chart a fresh paradigm, offering promising therapeutic avenues for cervical cancer management, with an emphasis on targeting *IMP3* and its associated metabolic accomplices.

## Figures and Tables

**Table 1:** Primer sequence of examined genes in our study.

Gene	Forward	Reverse
*GLUT1*	AAGTCCTTTGAGATGCTGATCCT	AAGATGGCCACGATGCTCAGATA
*GLUT4*	CCATCCTGATGACTGTGGCTCT	GCCACGATGAACCAAGGAATGG
*PDK1*	CACCGAGCTGCTGAAGAACT	TCCAGGTCCACAGCATCTTC
*PDK4*	CGGTCTCGAGAGAAAATGCATGTGAAAG	TTCCCGGGGGTAAAGGCGGCCCCG
*HK2*	TGGAGCCACCACATCAAAGA	CAGCGGTACAGGGTCTTGAT
*MCT4*	GCTGGTGCTGGTGTTCTCTT	CAGCCACAGCCACAGATACA
*TOP2A*	AGGATTCCGCAGTTACGTGG	CATGTCTGCCGCCCTTAGAA
*KIF15*	AAAACTGAGTTACGCAGCGTG	AGTTGCGAATACAGATTCCTGAG
*CENPF*	CTCTCCCGTCAACAGCGTTC	GTTGTGCATATTCTTGGCTTGC
*BUB1*	CGATGGTACCACCATGGACACCCCGG	CATAGCGGCCGCGCTTTTCGTGAACGC
*ACACA*	TTCACTCCACCTTGTCAGCGGA	GTCAGAGAAGCAGCCCATCACT
*FASN*	TTCTACGGCTCCACGCTCTTCC	GAAGAGTCTTCGTCAGCCAGGA
*SREBF1*	ACTTCTGGAGGCATCGCAAGCA	AGGTTCCAGAGGAGGCTACAAG

## Data Availability

The original contributions presented in this study are included in the article. Further inquiries can be directed to the corresponding author. The genetic information for all cell lines used in this study is publicly accessible in the Cellosaurus database. HeLa, SiHa, and C-33A correspond to accession numbers CVCL_0030, CVCL_0032, and CVCL_1094, respectively.

## Supplementary Materials

The following supporting information can be downloaded at: https://www.mdpi.com/article/10.3390/cimb47121014/s1.
