# Supplementary material for: Regulation of Energy Metabolism and Lipid Metabolites by IMP3 in Cervical Cancer
Source: Curr Issues Mol Biol. 2025 Dec 4;47(12):1014. doi: 10.3390/cimb47121014 (PMC12731437; doi:10.3390/cimb47121014)
Supplement: Supplementary file 1 [file cimb-47-01014-s001.zip › Sup Information-11.27.pdf]

SI results

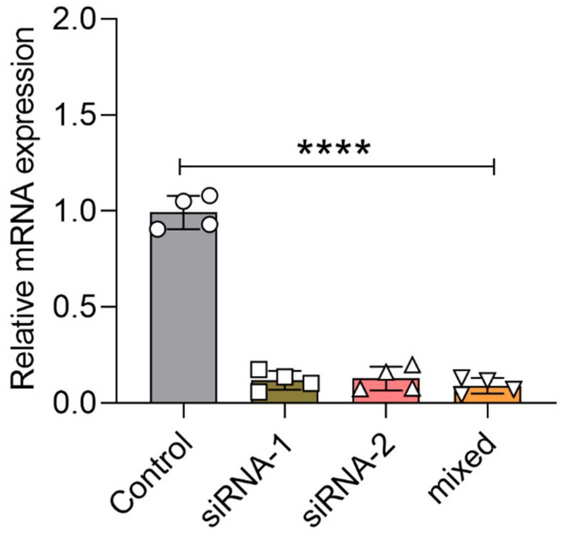

SI Figure S1. Quantitative measurement of IMP3 expression levels in Hela post shRNA transfection. Data were presented as Mean ± SD. Student's t-testing, **** $P < 0.0001$.

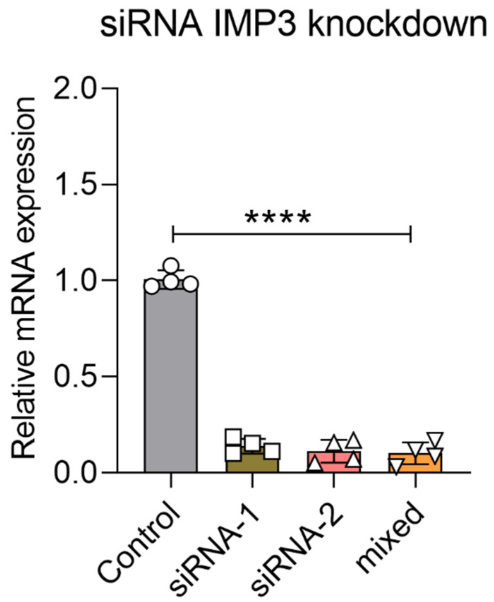

SI Figure S2. Quantitative measurement of IMP3 expression levels in Hela post siRNA transfection. Data were presented as Mean ± SD. Student's t-testing, **** $P < 0.0001$.

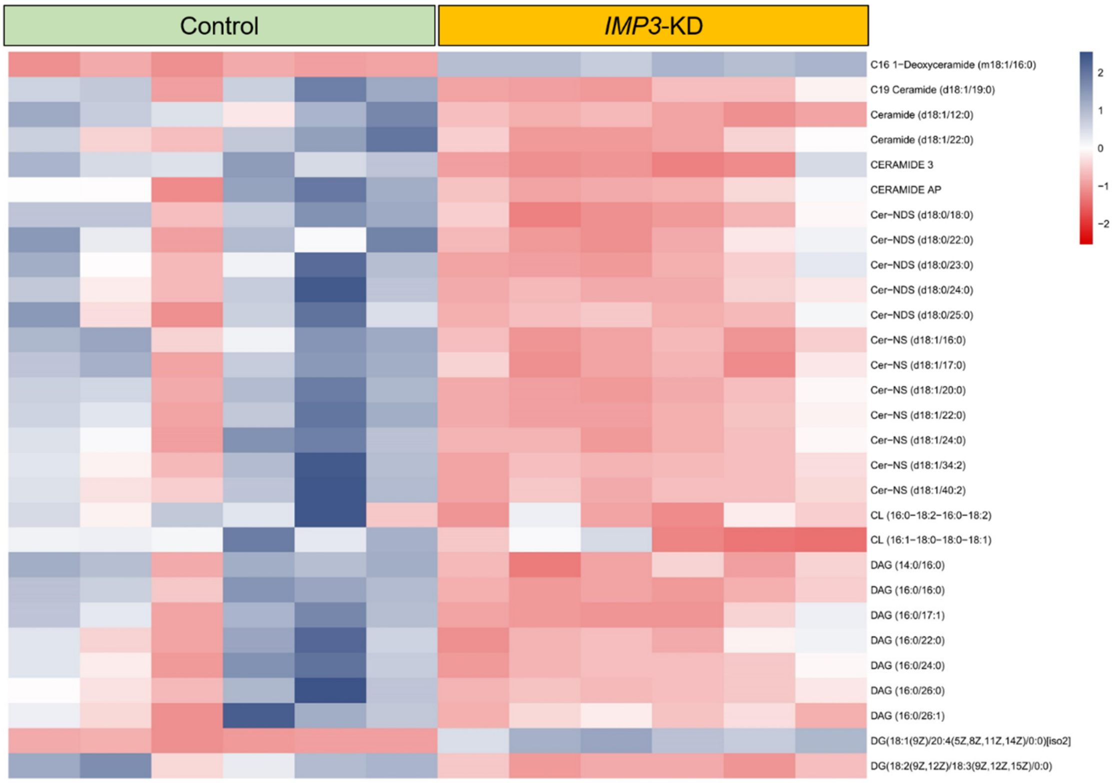

SI Figure S3. Profiles of lipid-related metabolites identified as significance. Blue color is attributed as the up-regulated levels, and red color is attributed as the down-regulated levels.

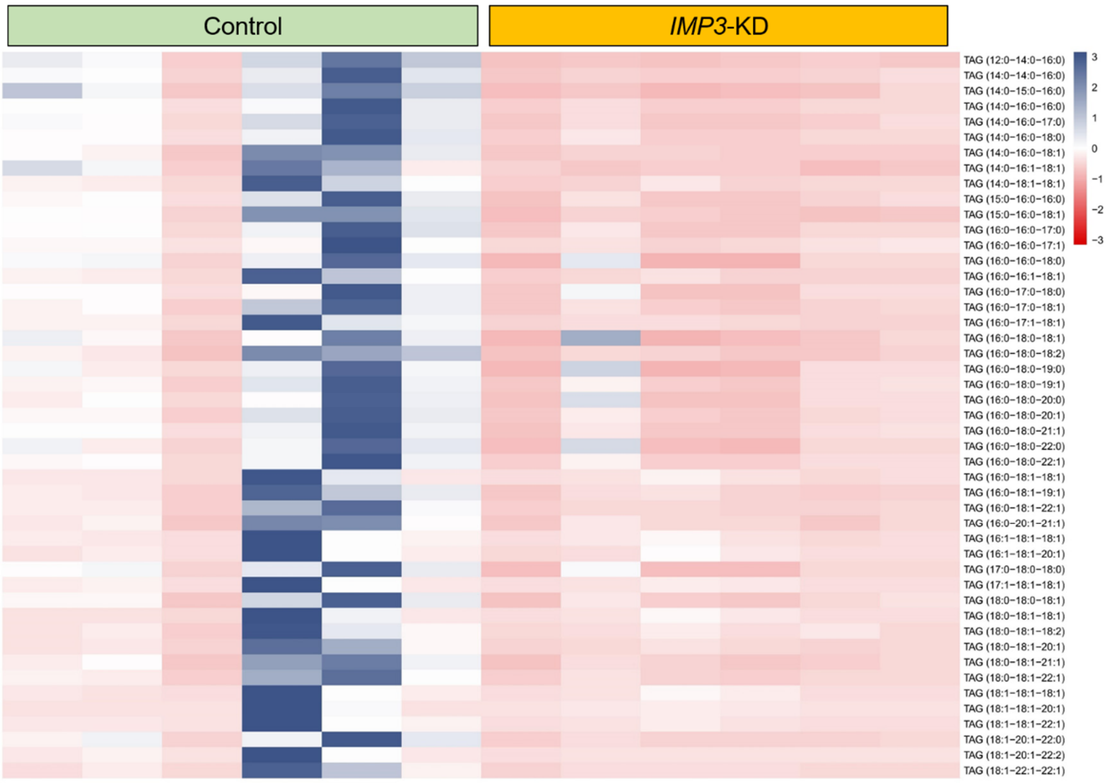

SI Figure S4. Heatmap illustrating the differentially expressed metabolites across *IMP3*-silenced Hela cells in TAG moieties. Each row represents a specific metabolite and each column represents an independent experimental group. The color scale represents z-scored

metabolite abundances with blue indicating high abundance and red indicating low abundance.

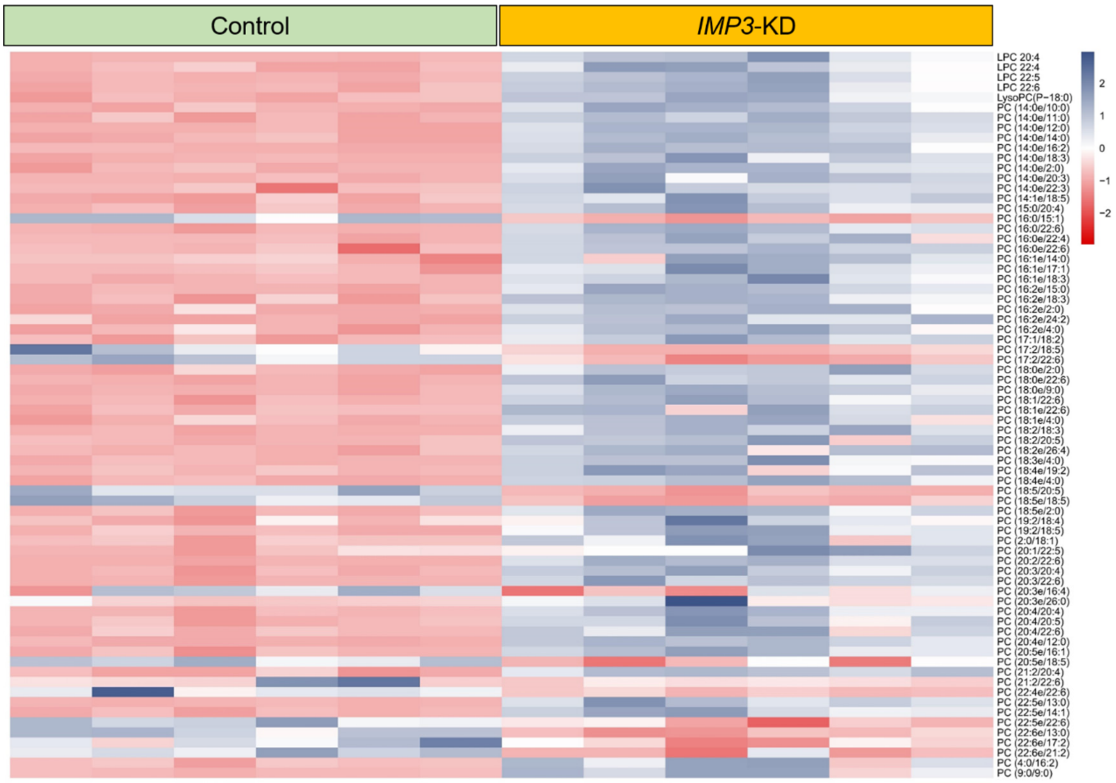

SI Figure S5. Heatmap illustrating the differentially expressed metabolites across *IMP3*-silenced Hela cells in LPC and PC moieties. Each row represents a specific metabolite and each column represents an independent experimental group. The color scale represents z-scored metabolite abundances with blue indicating high abundance and red indicating low abundance.

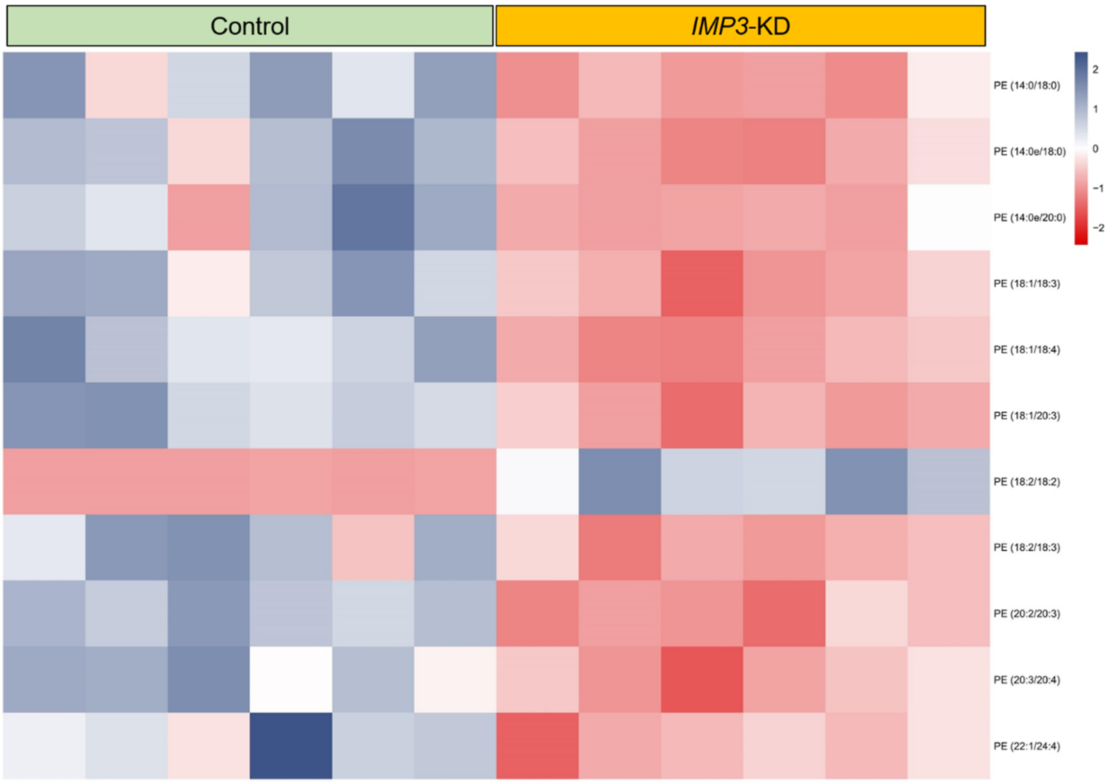

SI Figure S6. Heatmap illustrating the differentially expressed metabolites across *IMP3*-silenced Hela cells in PE moieties. Each row represents a specific metabolite and each column represents an independent experimental group. The color scale represents z-scored metabolite abundances with blue indicating high abundance and red indicating low abundance.

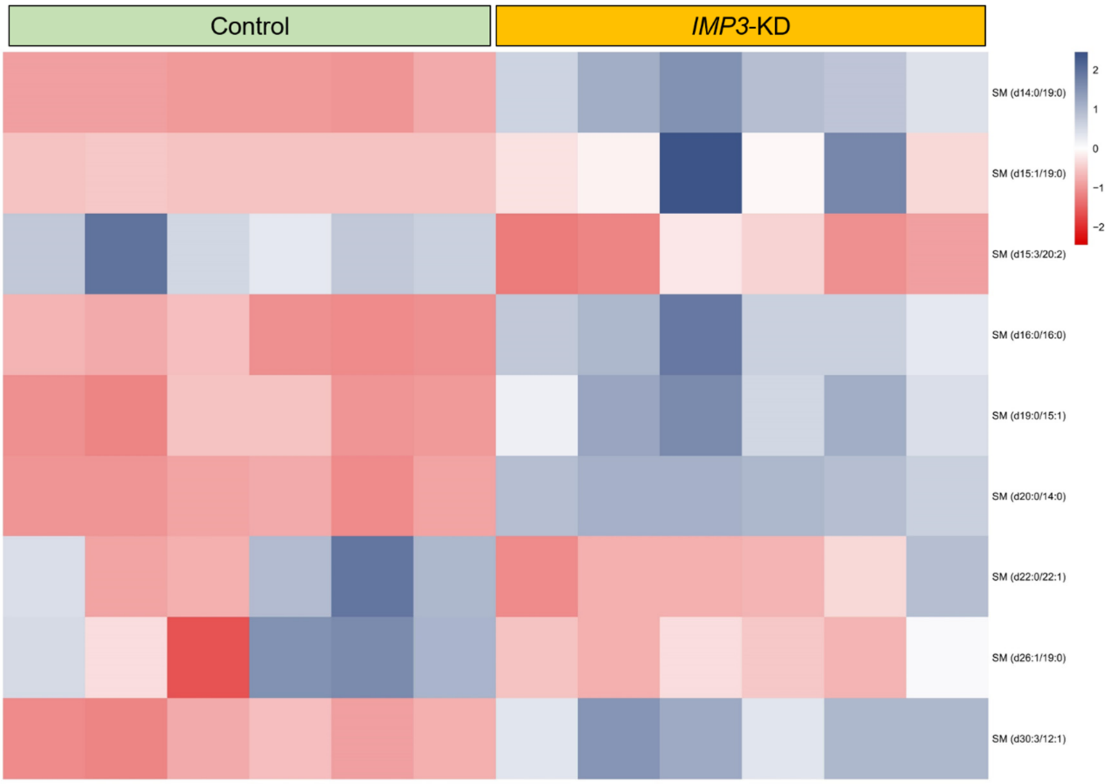

SI Figure S7. Heatmap illustrating the differentially expressed metabolites across *IMP3*-

silenced Hela cells in SM moieties. Each row represents a specific metabolite and each column represents an independent experimental group. The color scale represents z-scored metabolite abundances with blue indicating high abundance and red indicating low abundance.

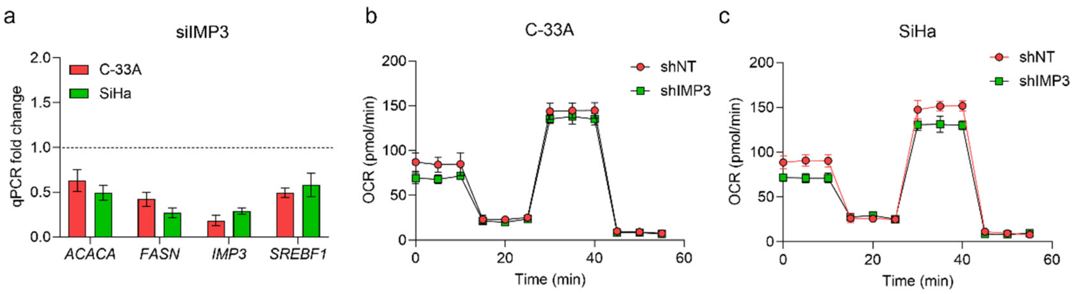

SI Figure S8. a, critical gene expression after knockdown in C-33A and SiHa cells. N=3 replicate per group. b-c, Seahorse XF Mito Stress Test OCR time-course in HeLa, SiHa, and C-33A cells with non-targeting (shNT) or IMP3 knockdown (shIMP3). Vertical dashed lines indicate injections (Oligomycin, FCCP, Rotenone/Antimycin A). Lines denote mean; shaded areas indicate SEM (n=6 wells per condition). Data are presented as Mean ± SD.

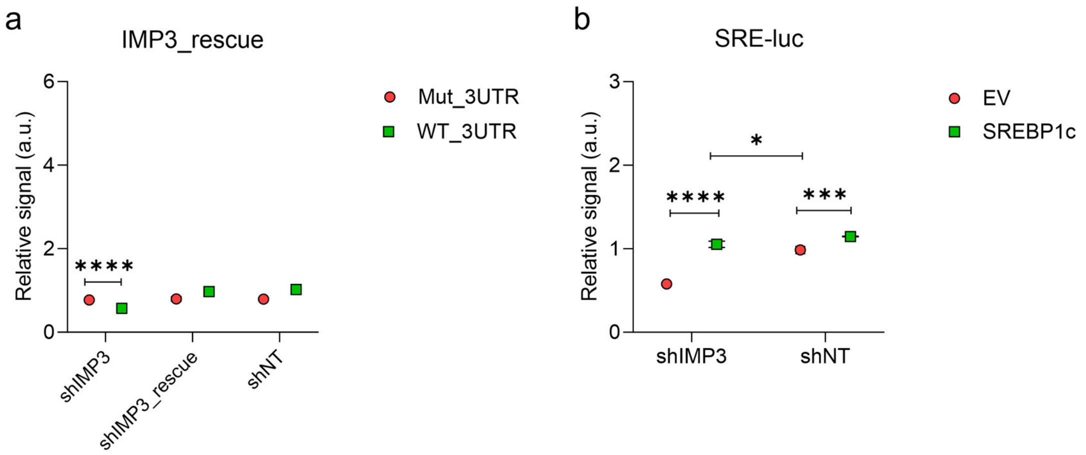

Supplementary Fig. S9. a. IMP3 promotes SREBF1 post-transcriptional output via the SREBF1 3′UTR. Dual-luciferase reporter assays in HeLa using Firefly reporters bearing wild-type SREBF1 3′UTR (WT_3UTR) or a motif-disrupted mutant (Mut_3UTR), co-transduced with shNT or shIMP3, with or without RNAi-resistant IMP3 re-expression (IMP3-res). b. IMP3 controls lipogenic transcriptional output *via* SREBP1c. SRE-luc reporter activity in HeLa under shNT or shIMP3, with or without SREBP1c ORF overexpression. Firefly was normalized to Renilla. Bars show mean ± s.d. (n = 3 biological replicates; technical triplicates averaged).

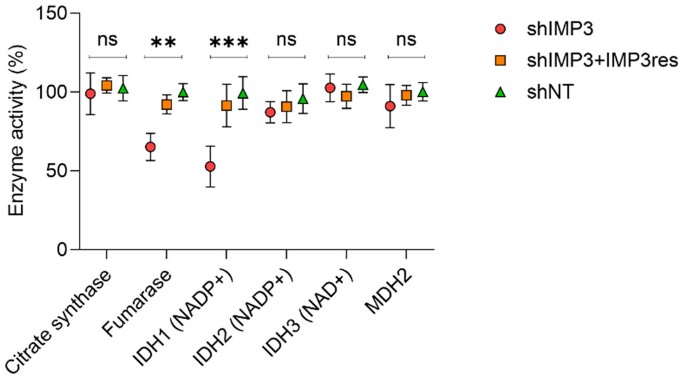

Supplementary Figure S10. Enzymatic activity pinpoints IDH1 and fumarase as rate-limiting nodes upon IMP3 loss. Bar plots show specific activities (nmol min^{-1} mg^{-1} protein) of IDH1 ($NADP^+$), IDH2 ($NADP^+$), IDH3 (NAD^+), fumarase, MDH2, and citrate synthase in HeLa cells transduced with shNT, shIMP3, or shIMP3 + IMP3^res (RNAi-resistant rescue). Data are mean ± s.d. (n = 5 biological replicates). Brackets indicate planned comparisons (KD vs NT; Rescue vs KD) analyzed by two-sided Welch's t-tests with Benjamini–Hochberg FDR correction; FDR-adjusted P values are annotated in-figure.
